# Forecasting influenza-like illness dynamics for military populations using neural networks and social media

**DOI:** 10.1371/journal.pone.0188941

**Published:** 2017-12-15

**Authors:** Svitlana Volkova, Ellyn Ayton, Katherine Porterfield, Courtney D. Corley

**Affiliations:** Data Sciences and Analytics Group, Computing and Analytics Division, National Security Directorate, Pacific Northwest National Laboratory, Richland, WA, United States of America; Georgia State University, UNITED STATES

## Abstract

This work is the first to take advantage of recurrent neural networks to predict influenza-like illness (ILI) dynamics from various linguistic signals extracted from social media data. Unlike other approaches that rely on timeseries analysis of historical ILI data and the state-of-the-art machine learning models, we build and evaluate the predictive power of neural network architectures based on Long Short Term Memory (LSTMs) units capable of nowcasting (predicting in “real-time”) and forecasting (predicting the future) ILI dynamics in the 2011 – 2014 influenza seasons. To build our models we integrate information people post in social media e.g., topics, embeddings, word ngrams, stylistic patterns, and communication behavior using hashtags and mentions. We then quantitatively evaluate the predictive power of different social media signals and contrast the performance of the-state-of-the-art regression models with neural networks using a diverse set of evaluation metrics. Finally, we combine ILI and social media signals to build a joint neural network model for ILI dynamics prediction. Unlike the majority of the existing work, we specifically focus on developing models for local rather than national ILI surveillance, specifically for military rather than general populations in 26 U.S. and six international locations., and analyze how model performance depends on the amount of social media data available per location. Our approach demonstrates several advantages: (a) Neural network architectures that rely on LSTM units trained on social media data yield the best performance compared to previously used regression models. (b) Previously under-explored language and communication behavior features are more predictive of ILI dynamics than stylistic and topic signals expressed in social media. (c) Neural network models learned exclusively from social media signals yield comparable or better performance to the models learned from ILI historical data, thus, signals from social media can be potentially used to accurately forecast ILI dynamics for the regions where ILI historical data is not available. (d) Neural network models learned from combined ILI and social media signals significantly outperform models that rely solely on ILI historical data, which adds to a great potential of alternative public sources for ILI dynamics prediction. (e) Location-specific models outperform previously used location-independent models e.g., U.S. only. (f) Prediction results significantly vary across geolocations depending on the amount of social media data available and ILI activity patterns. (g) Model performance improves with more tweets available per geo-location e.g., the error gets lower and the Pearson score gets higher for locations with more tweets.

## Introduction

Every year there are 500,000 deaths worldwide attributed to influenza including 30,000 – 50,000 deaths in the US [1]. The Centers for Disease Control and Prevention (CDC) reports weekly on the level of confirmed influenza and influenza-like illnesses (ILI) seen year round in hospitals and by doctor visits that are used to monitor the spread and impact of influenza. However, by the time the CDC data is released, the information is already several weeks old. To overcome this, researchers explored alternative data sources for monitoring influenza and ILI dynamics in real time including web queries [2], Wikipedia logs [3, 4], microblogs [5] and social media platforms, e.g., Twitter [6–9], as a way to enhance predictive ability for health officials when looking at influenza infection rates.

Researchers theorized that the most valuable impact alternative data sources [10, 11], e.g., Twitter, can make is by reducing the error in influenza predictions during the weeks the influenza infection rates are under revision by the CDC [7]. Indeed, they have shown through the use of basic linear autoregressive models that a combined model of Twitter and ILI data outperforms a similar model of only ILI data. These promising results give motivation for introducing richer models into this prediction task. The work done by [8] incorporates and experiments with several machine learning ensemble methods to forecast ILI dynamics. Using these models, they are able to accurately predict ILI activity for up to two weeks. However, they only explored basic bag-of-word features extracted from tweets. Similar to [9], we argue that to effectively utilize social media data, the existing natural language processing (NLP) techniques need to be improved or new methods developed in order to extract richer meaning from tweets. Furthermore, researchers advocate the use of Twitter as a way to supplement customary influenza monitoring systems to make accurate predictions [6, 12].

Following prior advances on infectious disease surveillance using social media data [7, 8], we made use of large amounts of public Twitter data – 171M tweets collected between 2011 – 2014. We considered this data as a real-time source of information in order to forecast ILI activity estimates—the total number of people seeking medical attention with ILI symptoms. We specifically focused on military populations and collected ILI activity data and Twitter data from fine-grained geolocations – 25 in the US and 6 international from 2011 to 2014.

To the best of our knowledge, this is a pioneer study that takes advantage of and evaluates the predictive power of Recurrent Neural Networks (RNNs) to predict ILI dynamics [13]. Moreover, unlike any previous work, the proposed models rely on different social media signals including lexical, stylistic, topics, emotions and opinions, and communication behavior patterns extracted from user tweets, and contrast model performance learned from social media data with models learned from ILI historical data. In addition, we contrast neural network model performance for nowcasting and forecasting ILI dynamics with the machine learning approaches explored by the current state-of-the-art approach [8]. More specifically, this work aims to answer several research questions:

- RQ1: What machine learning models yield the best ILI predictions, e.g., the state-of-the-art regression models or neural networks?
- RQ2: What signals from social media are the most predictive of ILI dynamics, e.g., word ngrams, stylistic, communication behavior, topics, or embeddings?
- RQ3: How accurately can we predict location-specific weekly ILI dynamics? How does prediction accuracy vary across locations in a nowcasting vs. forecasting setting (making predictions up to several weeks in advance)? How does model performance depend on the amount of social media data available per location?

We started our analysis by running machine learning models for ILI dynamics prediction for 2011 – 2014 on a sample dataset (4M tweets) for six geolocations to determine the best performing models and the most predictive social media signals. We found that language (word unigrams and embeddings) and communication behavior features (hashtags and mentions) are more predictive of ILI dynamics than stylistic signals extracted from social media communications. Moreover, we found that location-specific models outperform location-independent models and that prediction results vary significantly across geolocations.

We then applied the best performing models—LSTMs and social media features—and combined tweet and communication behavior signals to forecast ILI dynamics for 31 geolocations (171M tweets). We demonstrated that social media signals used to learn LSTM models yield comparable performance to ILI historical data. Thus, signals from social media can be potentially used to accurately forecast ILI dynamics for the regions where ILI historical data is not available. Moreover, we showed that neural network models trained on combined ILI and social media signals significantly outperform models that rely solely on ILI historical data.

We anticipate the proposed neural network models in combination with social media signals will enable surveillance epidemiologists to perform planetary-scale health monitoring, detect potential public health threats and capture early-level warnings for epidemics. Moreover, our approach is generalizable to other infectious diseases and social media platforms and can advance the existing disease surveillance approaches for E. coli [14], ebola [15, 16], and cholera [17]. Social media resources could be combined with other data streams [18] e.g., restaurant reservations [19], news sources [20], and pharmacy sales [21].

## Materials and methods

In this section, we present ILI-related clinical visit data across 31 geolocations and describe our social media data collection and sampling procedure. We then outline our experimental setup for two prediction tasks—nowcasting (predicting this week’s ILI rates) and forecasting (predicting ILI activity one and two weeks in advance), describe machine learning models, social media predictors, and evaluation metrics.

### ILI-Related clinical visit data

The ILI clinical data consists of the number of visits to a Defense Medical Information System (DMIS) Identifier (ID) location for symptoms identified as influenza-like illness (ILI) based on the International Statistical Classification of Disease and Health Related Problems (ICD) codes documented in the electronic patient record (Table 1). The DMIS ID facility types identified as reporting ILI symptoms in patients are hospitals, clinics, administration, and dental offices. The patients who visit these facilities include active duty, reserve, and retired members along with their dependents, and cadets, recruits, and applicants for active duty from the army, navy, marine corps, coast guard, air force, NOAA, and other public health services. This military health data was collected from 31 specific locations (25 U.S. and 6 international). Each location comprised all DMSID IDs within a 25-mile radius around military bases (mean 7 IDs, range 2-19 IDs). The percent of ILI visits to total visits was used in the subsequent analyses (mean 3.6%, range 1.1-9.5%) and is defined as weekly location-specific ILI visit proportions:

$$\begin{array}{c} ILI=\frac{＃\;\text{of weekly ILI visits per location}}{＃\;\text{of total weekly visits per location}} \end{array}$$ (1)

**Table 1:** The ICD-9 codes used to describe ILI symptoms (NOS = Not otherwise specified).

ICD9	Code Description for ILI Symptoms
79.99	Viral infection, NOS
382.9	Unspecified otitis media
460	Acute nasopharyngitis (common cold)
461.9	Acute sinusitis unspecified
465.8	Acute upper respiratory infections of other multiple sites
465.9	Acute upper respiratory infections of unspecified sites
466	Acute bronchitis
486	Pneumonia organism unspecified
487	Influenza with pneumonia
487.1	Influenza with other respiratory manifestations
487.8	Influenza with other manifestations
488	Influenza due to identified agent
490	Bronchitis, NOS
780.6	Fever
786.2	Cough

**Fig 1:**
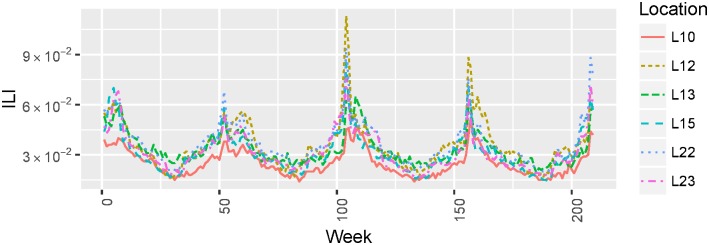
Location-specific ILI dynamics. Weekly ILI proportions between 2011 and 2014 for six example geolocations.

In Fig 1 we present weekly ILI proportion dynamics between 2011 and 2014 for six example geolocations e.g., L10, L12 etc.

We focused on ILI (influenza-like illness) predictions rather than influenza Virology data (which measures lab-confirmed cases of influenza) because: first, Virology data is not always considered to be a good predictor of ILI, and, second, it tends to be even more delayed than ILI data due to time spent on laboratory testing [22, 23].

### Twitter dataset

Twitter data, acquired from a social media vendor and through the public API, was anonymized for usernames, user IDs, and tweet IDs based on a rigorous procedure, i.e., a state-of-the-art encryption algorithm. To ensure the privacy of all users in our sampled datasets, our analysis was based only on completely anonymized data and our findings are reported on an aggregate rather than individual level. This study was approved by our institutional review board (IRB).

**Fig 2:**
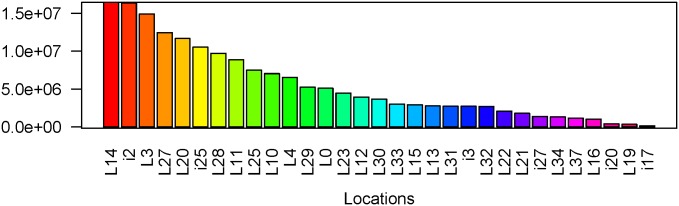
Tweet distribution across geolocations. The number of tweets collected within a 25-mile radius of military installations for 31 geolocations.

We collected geo-tagged tweets within a 25-mile radius of 25 military locations in the U.S. and six international locations (shown as *L* and *i* respectively on Fig 2) following standard practices on extracting geolocation coordinates from user meta-data [24]. The timeframe for collected tweets ranges between January 2011 to December 2014. Our large Twitter sample includes 171,027,275 tweets produced within a 25-mile radius across 31 military locations. We report tweet distribution for each military location in Fig 2.

For a subset of our experiments, we subsampled 2,169 users from six geolocations (CA, NC, TX) who explicitly reveal their military affiliation by mentioning military-specific keywords, e.g., *military, corporal, army brat,* etc. in their user profile data from the original dataset of 171 million tweets. We used the Twitter API to collect up to 3200 of their tweets. The total number of tweets in a sample is 4,029,715 including *L*12 = 381, 178, *L*15 = 467, 509, *L*23 = 607, 461, *L*22 = 578, 843, *L*10 = 378, 837, and *L*3 = 1,615,887.

### Models

We focus on two prediction tasks—nowcasting (predicting current week ILI rates) and forecasting (predicting ILI activity several weeks in advance). We define our nowcasting task as predicting weekly ILI proportions for the current week *Y*_*t*_*i*__ using data from the time period *X*_[*t*_*i*−*k*_, *t*_*i*−1_]_, where *k* is the window size between one and four weeks. We define our forecasting task as predicting weekly ILI proportions at week *Y*_*t*_*i*+1__ or *Y*_*t*_*i*+2__ (one or two weeks in advance, respectively) using data from the time period *X*_[*t*_*i*−*k*_, *t*_*i*_]_, inclusive.

#### Baseline models

We take advantage of different regression models previously used for predicting ILI dynamics [8] e.g., Support Vector Machine (SVM) and AdaBoost. We also experimented with Linear Regression with Ridge and Lasso regularization, however these models yielded the lowest performance and were excluded from our analysis. We experiment with two SVM models: one model with a linear kernel and the other with a radial basis function (RBF) kernel. However, unlike previous work that only relied on word ngram features extracted from tweets [7], we experiment with and contrast the predictive power of lexical, stylistic, and communication behavior predictors.

#### Neural network models with long short-term memory layers

Long short-term memory (LSTM) is a recurrent neural network with a built in memory cells to store information and exploit long range context, surrounded by gating units that can reset, read, and write information [25]. LSTMs have been successfully used for sequence modeling e.g, speech recognition, language modeling, translation, image captioning. LSTMs are computationally more powerful than other sequence models e.g., Hidden Markov Models with no continuous internal states, feedforward networks, and SVMs with no internal states at all.

**Fig 3:**
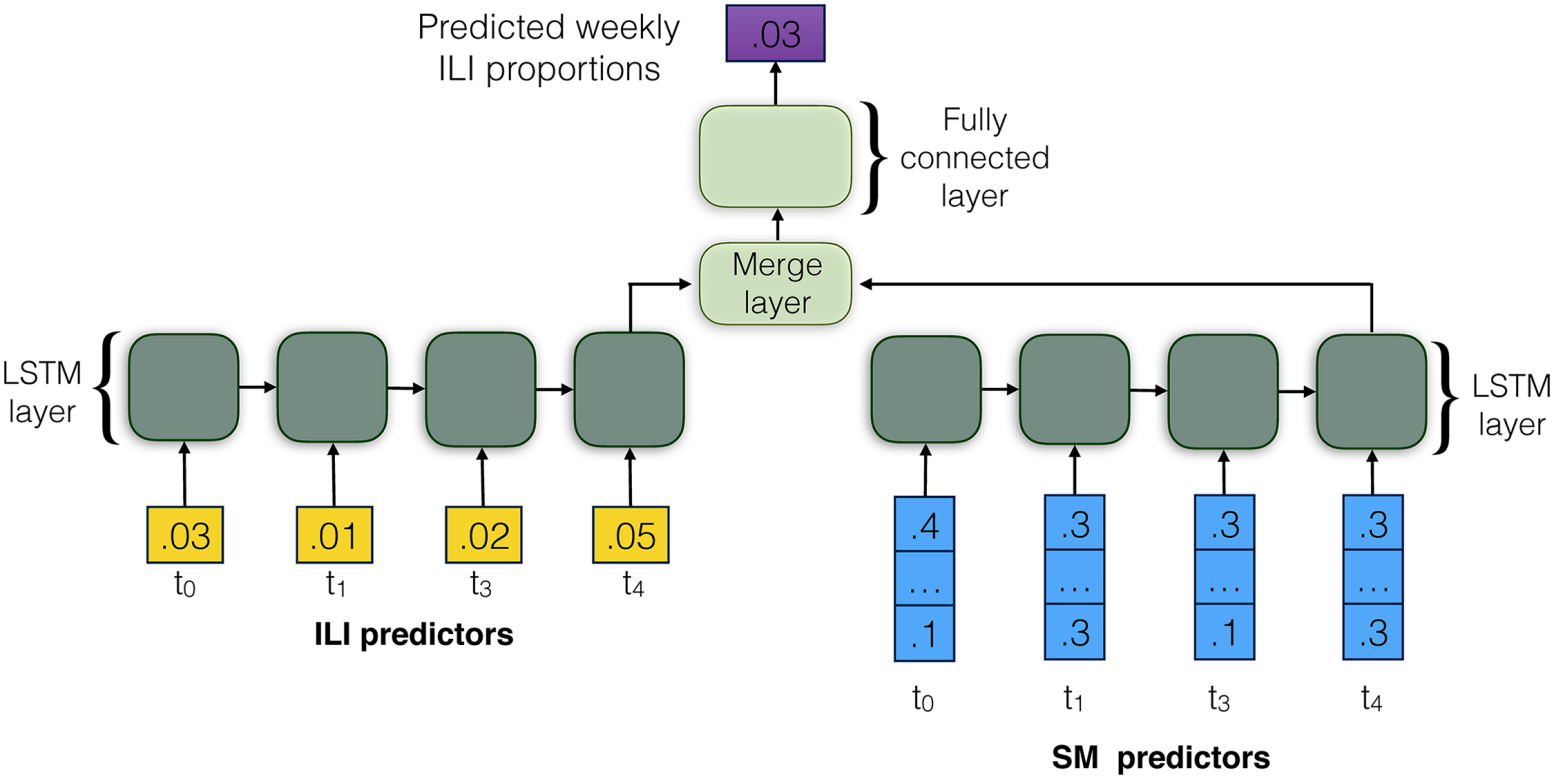
Diagram of a two-branch neural network model for ILI dynamics prediction. The model combines ILI historical estimates encoded using one LSTM layer on the left and social media predictors (ILI + SM) encoded using another LSTM layer on the right to forecast ILI dynamics in weeks.

For our experiments, we implement multiple neural network architectures that rely on LSTM layers in keras for regression to forecast weekly ILI proportions. To combine ILI historical data and social media signals we rely on a two-branch neural network architecture presented in Fig 3. For this combined SM and ILI setting, we add a merge layer before the fully connected layer. If we only rely on ILI historical data, we use the left branch of the network for ILI forecasting followed by a fully connected layer. We initialize the network with one input node per timestep/week (for ILIOnly) and a single output node, using the raw scalar output as the predicted value for regression. Likewise, if we only rely on social media signals, we use the right branch for ILI forecasting, followed by a fully connected layer. For social media signals (for SM Only), we initialize the network with 10k-dimensional vectors of word-level weekly-normalized statistics (focusing on the most frequent ngrams), 1K-dimensional pre-trained embedding vectors [26], and 2K-dimensional topic vectors [27]. While relatively simple (e.g., single rather than multilayer), the above described neural network architectures permit relatively short training time, and, thus, a scalable framework for even larger datasets.

Let **X**_*t*_ denote a matrix of training instances at time *t* that could be either weekly ILI proportions at time *t* (in the left branch of the network) or k-dimensional vectors of word-level weekly-normalized statistics (in the right branch of the network), or both. Let **I**_*t*_, **F**_*t*_, **C**_*t*_, **O**_*t*_, **M**_*t*_ denote input, forget and output gates, cell and hidden states of the LSTM at time *t*. Cells within LSTM layers of size 2,048 in both branches of the network are described mathematically below:

$$\begin{array}{c} \text{Input}\;\text{gate:}\;I_{t}=\sigma \left(X_{t}\cdot W_{xi}+M_{t-1}\cdot W_{hi}+C_{t-1}\cdot W_{ci}+b_{i}\right) \end{array}$$ (2)

$$\begin{array}{c} \text{Forget}\;\text{gate:}\;F_{t}=\sigma \left(X_{t}\cdot W_{xf}+M_{t-1}\cdot W_{hf}+C_{t-1}\cdot W_{cf}+b_{f}\right) \end{array}$$ (3)

$$\begin{array}{c} \text{Cell}\;\text{state:}\;C_{t}=F_{t}•C_{t-1}+I_{t}•tanh\left(X_{t}\cdot W_{xc}+M_{t-1}\cdot W_{hc}+b_{c}\right) \end{array}$$ (4)

$$\begin{array}{c} \text{Output}\;\text{gate:}\;O_{t}=\sigma \left(X_{t}\cdot W_{xo}+M_{t-1}\cdot W_{ho}+C_{t}\cdot W_{co}+b_{o}\right) \end{array}$$ (5)

$$\begin{array}{c} \text{Hidden}\;\text{state:}\;M_{t}=O_{t}•tanh\left(C_{t}\right) \end{array}$$ (6)

where (*σ*) denotes the sigmoid activation function, (⋅) indicates matrix multiplication and (•) indicates component-wise multiplication. W_*i_, W_*f_, W_*c_, W_*o_ are parameter matrices for gates learned during training. LSTM training is performed using backpropagation [28] with batch size of 16 using Adam optimizer (per-parameter adaptive learning rate method) to stabilize parameter updates to minimize mean squared error loss. All models are trained for 50 epochs. Note, no activation function is used for the dense output layer (fully connected layer as shown in Fig 3) because it is a regression task and we are predicting numerical ILI values directly without transform.

Our neural network model predicts the ILI value at time *t* in the time series by training with the data from a 4-week period prior to the *t* step. We experiment with forecasting and predict ILI values 1 to 2 weeks in advance using the 4-week sliding window. We perform this experiment using different types of features described below as the endogenous variables and weekly ILI proportions (Eq (1)) as the exogenous variable. We extract historical signals from ILI data (ILI Only), Social Media data (SM Only) or both ILI and Social Media data (ILI + SM).

We train our models independently for each location shown in Fig 2. We evaluate nowcasting experiments using 4-fold cross validation (2001 – 2014) for six example locations and 3-fold c.v. (2012 – 2014) for 31 locations. For the forecasting experiments, we train models for all years prior to 2014 (2011 – 2012 for six locations and 2012 – 2013 for 31 locations) and make predictions for 2014 season. We contrast the LSTM’s performance trained on ILI and social network data with the baseline AdaBoost and SVM regressors. We also compare location-specific models with a joint location-independent model trained on all locations jointly.

### Predictors

We extract different types of features (predictors) from tweets to train machine learning models for ILI activity prediction. These include: word ngrams (unigrams, bigrams, trigrams), term frequency-inverse document (TFIDF) scores [29], LDA topics [27], text embeddings [26], hashtags, mentions, and communication behavior features [30]. Before feature extraction, we pre-processed the data—tokenized, lowercased, removed punctuation, URLs, retweets, numbers, infrequent tokens, e.g., with frequency less than five in our Twitter corpus, and masked hashtag and mention symbols.

- **Tweets** From the cleaned tweets, we extracted unigrams, bigrams, trigrams, and TFIDF text representations. Our vocabulary size was proportional to the number of tweets from a given location and ranged from about 100K ngrams for locations with fewer tweets to 300K ngrams for locations with many more tweets.
- **Tweets and Network** In addition to text features we extracted hashtags and mentions (aka communication behavior features) exclusively as our ngram tweet representations ignored hashtags and mentions. Like n-gram features, we restricted these network predictors to a minimum frequency of 5 occurrences. Likewise, this vocabulary size was not fixed, but ranged depending on the location from 25K to 50K.
- **Topics** We learned topics discussed in tweets using Latent Dirichlet Allocation proposed by [27] on an independent sample of one million tweets. We tuned the number of topics *t* = [50, …, 1000] and Dirichlet priors *α* and *β*. We found that the optimal values of priors are *α* = 0.1 and *β* = 0.005, and topics *t* = 1000 by maximizing log-likelihood on a development subset of tweets. We converted tweets into topic vectors by replacing words with the corresponding topic assignments.
- **Text Embeddings** We relied on text embeddings obtained using the Word2Vec model developed by [26] implemented in gensim [31]. We learned embeddings on the same corpus of one million tweets as LDA topics. After learning embeddings—50-dimensional vector representations—we assigned words to clusters by measuring cosine similarity between pairs of word embeddings, and computed clusters using spectral clustering over a word-to-word similarity matrix. Similar to topics, we converted tweets into embedding vectors by replacing words with the corresponding cluster assignments.
- **Stylistic** We aggregated all tweets per week and calculated 14 stylistic features including the average tweet length in words and characters; retweet, uppercased *COOL*, elongated *Yaay* and repeated mixed punctuation *???!!!* rate; proportions of tweets that are retweets, contain URLs, mentions, hashtags, punctuation, and emotions; and mention, hashtag, and IRL rate per word.

Tweets, topics, and embeddings are inputs to the LSTM layer shown in Fig 3 on the right, also represented as an example **X**_*t*_ matrix of instances in Eqs (2)–(6). More precisely, **X**_*t*_ is either a sequence of 10k-dimensional vectors of word-level weekly-normalized statistics, or 1K-dimensional pre-trained embedding vectors, or 2K-dimensional topic vectors over time/window.

### Evaluation metrics

To evaluate the performance of our models and to compare against other work [8], we report four evaluation metrics: Pearson correlation (CORR), root mean squared error (RMSE), root mean squared percent error (RMSPE), and maximum absolute percent error (MAPE). These evaluation metrics were calculated for the time period January 1, 2011 to December 31, 2014. The definitions for all of the metrics are given below. We define *Y*_*t*_*i*__ to denote the observed value of the ILI proportion at time *t*_*i*_, ${\hat{Y}}_{t_{i}}$ denotes the predicted value by any model at time *t*_*i*_, $Y_{t_{i}}^{\prime }$ denotes the mean of the values of *Y*_*t*_*i*__, and $\hat{Y_{t_{i}}^{\prime }}$ denotes the mean of the values of ${\hat{Y}}_{t_{i}}$.

Pearson Correlation (CORR) measures the linear dependence between the predicted and observed values during the time period [*t*_1_, *t*_*n*_]:

$$\begin{array}{c} r=\frac{\sum_{i=1}^{n}\left(Y_{t_{i}}-Y_{t_{i}}^{\prime }\right)\left({\hat{Y}}_{t_{i}}-{\hat{Y}}_{t_{i}}^{\prime }\right))}{\sqrt{\sum_{i=1}^{n}{\left(Y_{t_{i}}-Y_{t_{i}}^{\prime }\right)}^{2}}\sqrt{\sum_{i=1}^{n}{\left({\hat{Y}}_{t_{i}}-{\hat{Y}}_{t_{i}}^{\prime }\right)}^{2}}} \end{array}$$ (7)

Root Mean Squared Error (RMSE) measures the difference between the predicted and observed values:

$$\begin{array}{c} RMSE=\sqrt{\frac{1}{n}\sum_{i=1}^{n}{\left(Y_{t_{i}}-{\hat{Y}}_{t_{i}}\right)}^{2}} \end{array}$$ (8)

Root Mean Squared Percent Error (RMSPE) measures the percent difference between the predicted and observed values:

$$\begin{array}{c} RMSPE=\sqrt{\frac{1}{n}\sum_{i=1}^{n}{\left(\frac{Y_{t_{i}}-{\hat{Y}}_{t_{i}}}{Y_{t_{i}}}\right)}^{2}}*100 \end{array}$$ (9)

Maximum Absolute Percent Error (MAPE) measures the magnitude of the maximum percent difference between the predicted and observed values:

$$\begin{array}{c} MAPE=(\max_{i}\frac{|Y_{t_{i}}-{\hat{Y}}_{t_{i}}|}{Y_{t_{i}}})*100 \end{array}$$ (10)

The evaluation metrics allow us to estimate model accuracy (Pearson correlation *r*, RMSE, RMSPE), robustness (MAPE), and the ability to predict upward and downward ILI tendency. All evaluation metrics were calculated for the time period from January 2011 to December 2014.

## Results

This section presents nowcasting and forecasting results for location-specific ILI dynamics prediction. We first focus on analyzing machine learning models and contrast different social media signals by training independent models on tweets (ngrams, TFIDF), text embeddings, and tweet and network (hashtags and embeddings) predictors for six example geolocations. We then take the best model and feature combinations and report ILI activity forecasting results for 31 geolocations. Finally, we investigate how model performance correlates with the number of social media posts available per location.

### RQ1: Contrasting predictive models

#### Nowcasting

Table 2 presents nowcasting (current week) prediction performance for three machine learning models—AdaBoost, SVM with a linear kernel, and LSTM models learned from ILI predictors and LSTM model learned from social media features—tweets, tweets and network, and text embeddings. The results are reported using four evaluation metrics—Pearson correlation (CORR), Root Mean Squared Error (RMSE), Root Mean Squared Percent Error (RMSPE), and Maximum Absolute Percent Error (MAPE) scores. These metrics were calculated for predictions over the time period from January 2011 to December 2014 using 4-fold cross validation. The results were averaged over six locations with the mean, minimum, and maximum values reported for each metric.

**Table 2:** ILI nowcasting results (current week) for six geolocations estimated using cross-validation over four years (2011–2014). Models: AdaBoost, SVM with a linear kernel, and LSTM. Metrics: Pearson correlation (CORR), RMSPE (%), MAPE (%), and RMSE. The highest performance results within each datatype are highlighted in bold.

	AdaBoost			SVM			LSTM		
Metric	Mean	Min	Max	Mean	Min	Max	Mean	Min	Max
**ILI Historical Data**									
CORR	0.81	0.43	0.92	**0.90**	0.85	0.99	0.86	0.84	0.87
RMSE	0.01	0.00	0.02	0.02	0.00	0.03	**0.01**	0.00	0.01
RMSPE	12.93	11.63	15.80	**10.31**	0.94	13.35	15.06	12.31	17.54
MAPE	42.59	23.81	68.91	**32.20**	4.30	41.89	53.04	42.14	65.01
**Social Media: Tweets Only (word bigrams)**									
CORR	0.47	0.27	0.65	0.19	0.15	0.44	**0.79**	0.66	0.85
RMSE	0.04	0.03	0.05	0.09	0.06	0.12	**0.01**	0.01	0.01
RMSPE	30.48	26.04	33.33	72.03	48.70	102.81	**29.52**	20.25	43.50
MAPE	81.02	59.18	112.64	129.74	100.00	219.87	**69.54**	45.05	111.35
**Social Media: Text Embeddings**									
CORR	0.60	0.30	0.76	**0.64**	0.51	0.77	0.22	0.02	0.35
RMSE	0.04	0.03	0.04	0.06	0.04	0.09	**0.01**	0.01	0.01
RMSPE	**29.92**	17.60	37.57	52.54	38.57	80.35	35.35	26.55	40.51
MAPE	**81.38**	42.10	130.21	114.62	81.66	188.84	89.62	61.86	111.74
**Social Media: Tweets and Network (hashtags)**									
CORR	0.61	0.45	0.73	0.58	0.41	0.71	**0.79**	0.64	0.86
RMSE	0.04	0.02	0.06	0.06	0.04	0.10	**0.01**	0.01	0.01
RMSPE	**31.81**	18.61	42.37	47.07	29.58	81.84	34.24	23.40	52.86
MAPE	83.97	45.10	107.60	116.39	74.64	209.14	**80.84**	50.84	135.99

As Table 2 shows, ILI historical signals yield better performance compared to social media signals (as has been shown earlier [8, 9]), with the SVM model producing the highest average CORR (*r* = 0.90), RMSPE (10.31%), and MAPE (32.2%). The best correlation per location was as high as 0.99. Thus, ILI predictors are very robust as indicated by MAPE and accurate as indicated by low average RMSE (0.01).

When we relied on no ILI historical data but only social media predictors to build models we found that LSTM models outperform other approaches in all metrics e.g., Pearson correlation (0.79), RMSE (0.01), RMSPE (29.52), and MAPE (69.54). We observed that out of all social media predictors tweets yield the highest performance compared to text embeddings and hashtags.

### RQ2: Evaluating predictive power of social media signals

**Fig 4:**
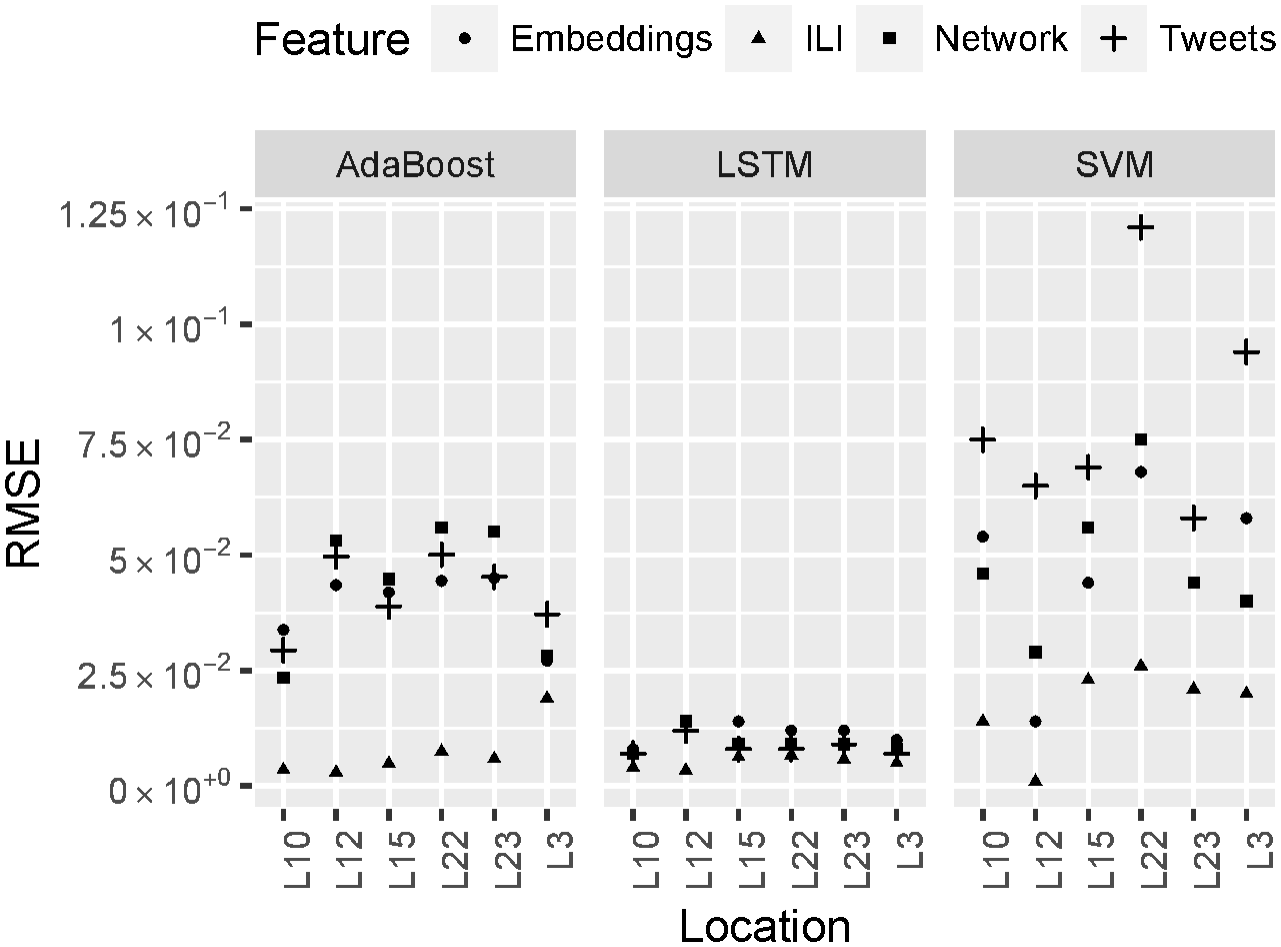
ILI nowcasting results for six geolocations from various social media signals estimated using cross-validation over four years (2011–2014). Predictive models—AdaBoost, SVM, and LSTM. Predictive features—ILI, Network, Tweets, and Embeddings. Evaluation metric—Root Mean Squared Error (RMSE).

Fig 4 reports detailed results for the current week ILI prediction across six geolocations obtained using three models—AdaBoost, SVM and LSTM, and four feature types—ILI, network, tweets, and embeddings. We observe that model performance significantly varies across locations e.g., RMSE is lower for L12 and L10, but 2.5 times higher for other locations; for the SVM model, RMSE is three time higher for tweets compared to ILI features; for the AdaBoost model social media signals yield 5 times higher RMSE than ILI features.

**Fig 5:**
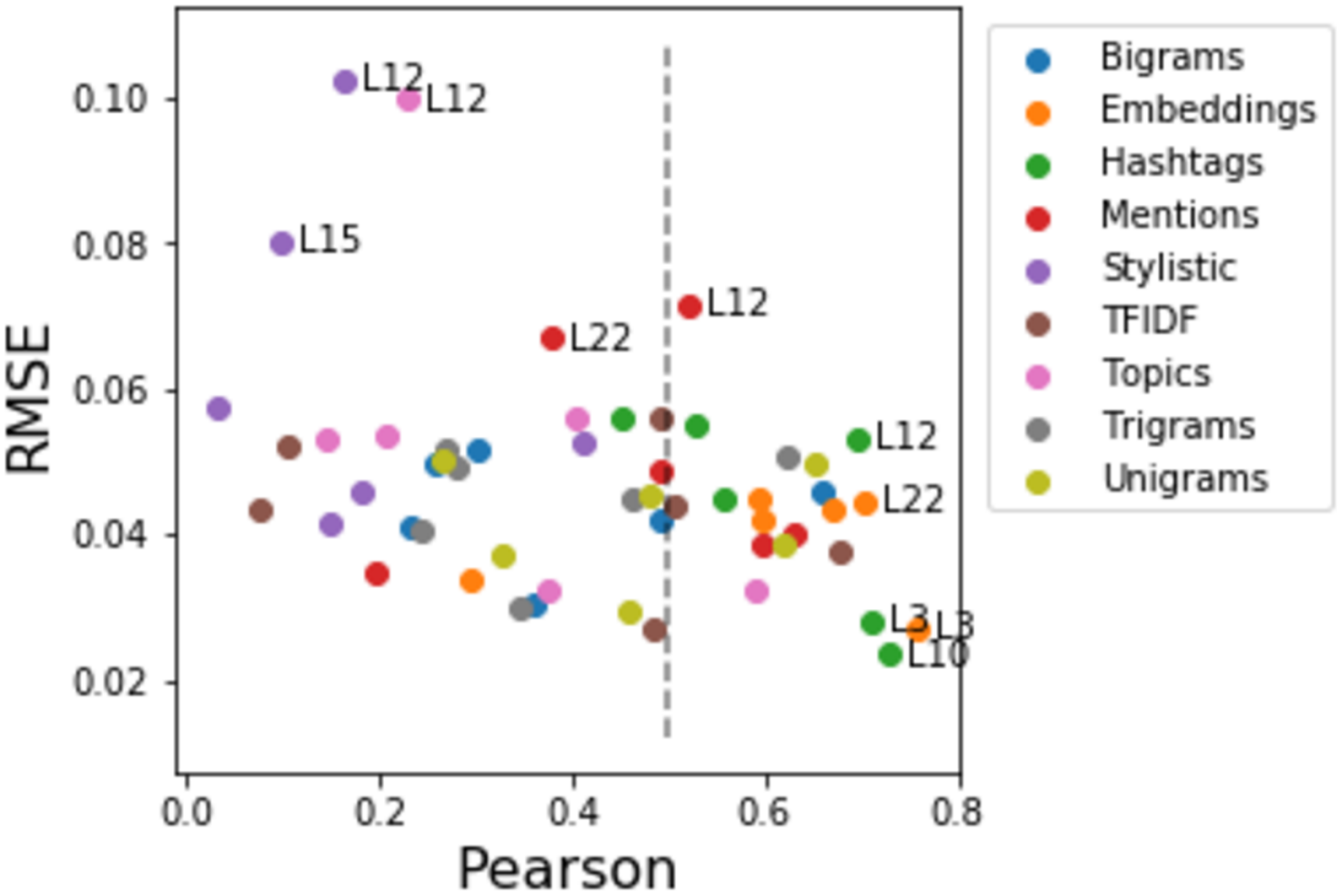
ILI nowcasting results for six geolocations obtained using SMOnly model trained on nine types of social media signals. We contrast Pearson and RMSE for six geolocations. Locations with labels show the best (embeddings, unigrams, hashtags and mentions), on the right from the dotted vertical line, and the worst (stylistic and topics) social media signals.

TFIDF, higher order ngrams, and stylistic features yield significantly lower performance as reported in Fig 5 compared to other types of social media predictors e.g., embeddings, and tweet and network signals. We found that embeddings, unigrams (tweets), hashtags and mentions (tweets and network) yield the highest Pearson and the lowest RMSPE compared to all other types of social media signals.

**Fig 6:**
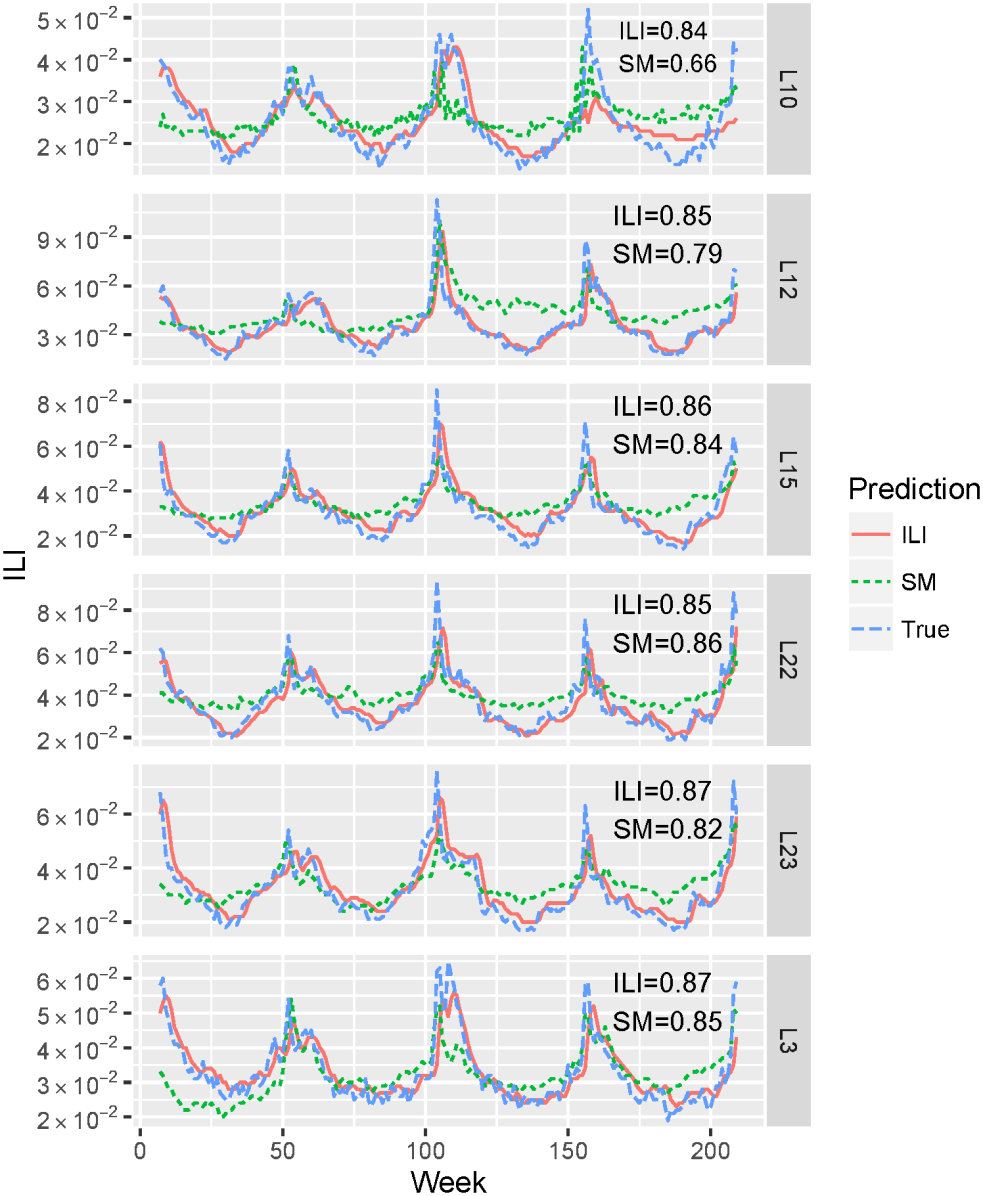
True vs. predicted ILI proportions (real-time current week estimates) as a function of time (2011–2014) for six geolocations. We plot true ILI proportions (True), predictions from social media (tweet and network) features (SM), and predictions from ILI historical data (ILI) obtained using LSTM model.

Real-time (nowcasting) predictions produced by the LSTM model learned from tweet and network (SM) signals are capable of predicting the timing and magnitude of yearly peaks as shown in Fig 6. Prediction performance varies across locations e.g., Pearson correlation is between *r*_*L*10_ = 0.66 and *r*_*L*22_ = 0.86 for social media signals and between *r*_*L*10_ = 0.84 and *r*_*L*22_ = 0.87 for ILI signals.

The results presented in Fig 6 qualitatively show the predictive power of social media features compared to ILI predictors. Overall, SM signals accurately track (the average Pearson correlation is 0.79) ILI proportions between 2011 and 2014 across six geolocations. Moreover, the fact that the difference in Pearson correlation between ILI and SM predictors is between 0.02 and 0.05 for four locations demonstrates the value of using solely social media data to predict ILI dynamics. This is especially valuable for the regions where historical ILI estimates are not available.

#### Forecasting

Table 3 presents ILI forecasts for one week in advance obtained using different models and social media predictors—tweets, topics, text embeddings, and network. First, as expected, the LSTM model can estimate ILI proportion for the current week with higher accuracy than forecasting ILI one week in advance. For example, the highest Pearson correlation obtained using LSTM model with SM data for the current week estimates is *r* = 0.79 vs. next week estimates *r* = 0.61, similarly for ILI data current week estimates *r* = 0.86 vs. next week estimates *r* = 0.84. We also observed RMSE for nowcasting is significantly lower than for forecasting one and two weeks in advance.

**Table 3:** ILI forecasting (one week) results for six geolocations. Models: AdaBoost, SVM with a linear kernel, and LSTM. Metrics: RMSPE (%), MAPE (%), and RMSE (% ILI). The best performing models within each data type are highlighted in bold.

	AdaBoost			SVM			LSTM		
Metric	Mean	Min	Max	Mean	Min	Max	Mean	Min	Max
**ILI Historical Data**									
CORR	0.87	0.78	0.91	**0.90**	0.85	0.99	0.84	0.79	0.89
RMSE	0.02	0.01	0.03	0.02	0.00	0.03	**0.01**	0.00	0.01
RMSPE	13.35	11.51	17.88	**10.43**	1.04	13.29	15.46	12.45	20.17
MAPE	43.61	23.87	79.53	**31.84**	4.50	40.23	49.07	39.83	65.42
**Social Media: Tweets Only (word unigrams)**									
CORR	0.53	0.41	0.69	0.23	0.11	0.46	**0.61**	0.54	0.64
RMSE	0.04	0.03	0.05	0.09	0.06	0.12	**0.01**	0.00	0.01
RMSPE	29.90	25.55	35.78	70.25	50.97	97.87	**20.43**	17.50	23.05
MAPE	73.42	52.72	98.11	127.99	100.00	207.35	**46.67**	37.31	54.26
**Social Media: Text Embeddings**									
CORR	0.61	0.46	0.76	**0.68**	0.56	0.77	0.18	0.08	0.29
RMSE	0.04	0.03	0.05	0.05	0.04	0.07	**0.01**	0.01	0.01
RMSPE	29.89	25.32	34.68	43.33	26.00	65.11	**29.13**	21.15	39.75
MAPE	83.20	69.60	105.26	94.04	52.36	145.65	**74.71**	47.92	108.58
**Social Media: Topics**									
CORR	0.43	0.34	0.61	**0.53**	0.45	0.66	0.21	0.16	0.27
RMSE	0.05	0.03	0.08	0.06	0.05	0.09	**0.01**	0.01	0.01
RMSPE	42.15	20.60	69.71	50.54	35.03	83.12	**28.80**	21.99	37.93
MAPE	116.73	47.86	192.48	112.85	70.33	191.04	**75.74**	51.50	103.99
**Social Media: Tweets and Network (hashtags)**									
CORR	0.54	0.43	0.64	**0.64**	0.58	0.73	0.59	0.53	0.66
RMSE	0.05	0.03	0.08	0.05	0.04	0.08	**0.01**	0.01	0.01
RMSPE	40.93	29.56	67.97	40.42	27.91	53.26	**26.63**	17.72	38.61
MAPE	112.70	79.76	182.72	99.02	69.56	131.91	**63.30**	38.54	97.03

Our key findings for six example geolocation are listed below.

- RQ1: LSTM models yield more accurate predictions compared to previously used AdaBoost or SVM models learned from social media signals.
- RQ2: Tweet and network signals are the most predictive signals within all other types of social media signals of ILI dynamics compared to stylistic, TFIDF, and higher order ngram features.
- ILI predictions significantly vary across locations. Model performance depends on the amount of social media data available and ILI dynamic complexity.
- Models for the current week estimates (nowcasting) are more accurate than models for one or two week forecasts.

### RQ3: Forecasting using ILI and social media data

In Tables 4–6 we report ILI prediction results (using Pearson correlation, RMSPE, and MAPE, respectively) for 31 geolocations. We train models using (1) ILI historical data, (2) the most predictive social media signals—tweets and network, and the best performing models—LSTM, and (3) joint ILI and SM signals. We train LSTM models on two season data (2012–2013) and test on the 2014 season.

**Table 4:** ILI predictions for 31 geolocations estimated using Pearson correlation for nowcasting (this week) and forecasting (one and two weeks). Neural network models are trained from ILI data only (ILI), social media data only (SM), or both ILI and SM data (ILI + SM). Locations are sorted by the amount of Twitter data available in a descending order (the first column is shown in millions). Locations with min and max correlations are underlined.

		This Week			One Week			Two Weeks		
Tweets		ILI	SM	ILI + SM	ILI	SM	ILI + SM	ILI	SM	ILI + SM
16.43	L14	0.86	0.97	0.97	0.73	0.94	0.94	0.55	0.88	0.92
16.38	i2	0.77	0.58	0.78	0.65	0.58	0.72	0.49	0.53	0.61
14.90	L3	0.84	0.94	0.96	0.72	0.90	0.93	0.61	0.84	0.89
12.45	L27	0.86	0.88	0.96	0.74	0.82	0.87	0.54	0.81	0.84
11.67	L20	0.70	0.88	0.91	0.25	0.74	0.69	0.01	0.59	0.62
10.53	i25	0.83	0.77	0.82	0.67	0.79	0.76	0.51	0.79	0.76
9.73	L28	0.87	0.90	0.96	0.75	0.82	0.88	0.54	0.79	0.84
8.87	L11	0.89	0.94	0.97	0.79	0.93	0.92	0.64	0.90	0.91
7.49	L25	0.86	0.91	0.96	0.76	0.86	0.93	0.63	0.87	0.93
7.04	L10	0.86	0.95	0.93	0.82	0.95	0.88	0.68	0.92	0.85
6.52	L4	0.86	0.95	0.97	0.79	0.93	0.95	0.69	0.91	0.90
5.25	L29	0.88	0.79	0.92	0.79	0.76	0.84	0.57	0.75	0.76
5.11	L0	0.75	0.61	0.89	0.69	0.56	0.82	0.58	0.65	0.79
4.48	L23	0.85	0.93	0.96	0.72	0.85	0.88	0.53	0.83	0.83
3.94	L12	0.90	0.84	0.95	0.82	0.90	0.91	0.69	0.95	0.89
3.67	L30	0.86	0.92	0.94	0.71	0.85	0.84	0.50	0.81	0.73
3.00	L33	0.87	0.87	0.94	0.76	0.82	0.89	0.54	0.77	0.84
2.92	L15	0.89	0.96	0.96	0.77	0.92	0.88	0.59	0.91	0.84
2.77	L13	0.78	0.81	0.92	0.57	0.65	0.75	0.33	0.59	0.55
2.73	L31	0.88	0.91	0.96	0.81	0.88	0.89	0.67	0.89	0.84
2.73	i3	0.59	0.54	0.66	0.40	0.47	0.47	0.24	0.43	0.33
2.71	L32	0.85	0.84	0.74	0.74	0.89	0.86	0.51	0.88	0.87
2.08	L22	0.83	0.90	0.95	0.71	0.83	0.86	0.54	0.83	0.77
1.80	L21	0.81	0.93	0.95	0.62	0.81	0.81	0.46	0.74	0.69
1.39	i27	0.86	0.63	0.84	0.79	0.74	0.82	0.61	0.80	0.72
1.33	L34	0.85	0.91	0.95	0.72	0.87	0.88	0.52	0.83	0.81
1.16	L37	0.83	0.57	0.85	0.75	0.45	0.75	0.61	0.38	0.59
1.04	L16	0.86	0.87	0.92	0.73	0.84	0.79	0.50	0.81	0.69
0.41	i20	0.88	0.37	0.81	0.77	0.22	0.63	0.65	0.06	0.33
0.35	L19	0.86	0.70	0.92	0.75	0.74	0.80	0.55	0.77	0.66
0.16	i17	0.80	0.38	0.68	0.59	0.32	0.55	0.38	0.33	0.37
**Mean**		**0.84**	**0.80**	**0.90**	**0.71**	**0.76**	**0.82**	**0.53**	**0.74**	**0.74**

**Table 5:** ILI prediction results for 31 geolocations estimated using RMSPE for nowcasting (this week) and forecasting (one and two weeks in advance). Locations with min and max correlations are underlined.

	This Week			One Week			Two Weeks		
	ILI	SM	ILI + SM	ILI	SM	ILI + SM	ILI	SM	ILI + SM
L14	40.2	48.8	38.7	45.3	65.5	32.4	61.8	74.9	30.2
i2	29.0	28.2	30.3	28.7	26.9	25.2	33.7	30.3	26.2
L3	39.7	41.7	55.4	53.5	73.9	40.6	52.4	68.6	53.6
L27	42.4	38.7	21.0	50.4	50.3	44.2	57.4	57.4	49.3
L20	50.2	55.7	48.7	65.6	59.8	67.1	61.2	57.4	51.2
Li25	79.1	53.9	54.0	77.4	54.4	75.1	95.2	70.1	73.4
L28	36.3	31.1	26.6	43.3	51.5	34.0	51.5	58.1	37.2
L11	37.3	50.0	31.9	42.6	71.7	45.6	57.7	55.9	47.0
L25	33.7	54.4	15.5	35.4	70.9	24.2	48.1	67.2	26.7
L10	38.8	40.7	33.4	38.3	37.7	33.6	49.7	54.6	29.9
L4	37.6	44.2	31.0	45.9	58.8	50.0	52.4	55.1	49.7
L29	35.0	45.1	42.1	46.0	49.2	40.2	54.6	48.7	43.3
L0	28.9	25.7	20.1	29.2	32.0	26.2	35.9	45.2	34.8
L23	38.8	62.0	30.0	45.3	74.2	40.2	51.8	65.6	45.0
L12	44.9	80.3	50.6	66.5	105.0	88.9	99.1	149.2	106.4
L30	48.0	62.8	35.1	54.6	75.7	48.9	63.5	75.1	60.4
L33	33.1	37.9	27.2	44.7	42.0	29.4	43.2	38.1	33.9
L15	56.9	99.5	40.5	62.2	107.5	71.9	76.8	154.6	91.0
L13	49.6	54.0	34.5	55.6	73.6	51.2	64.4	81.9	57.7
L31	39.7	70.6	29.3	44.5	79.1	38.9	48.1	71.5	57.8
i3	61.6	63.1	65.8	78.1	62.4	70.4	55.9	63.6	28.9
L32	43.1	97.4	88.3	57.1	97.8	74.6	71.9	141.7	97.1
L22	44.2	74.0	31.0	54.2	68.3	55.3	69.6	84.0	69.3
L21	49.7	83.5	37.7	65.0	68.7	59.5	77.0	90.0	75.7
i27	32.6	67.1	46.1	51.5	62.2	53.5	58.7	72.3	61.1
L34	29.2	36.8	22.6	38.6	38.4	35.0	49.8	39.9	44.6
L37	46.7	58.2	37.5	50.0	62.3	51.4	58.2	59.8	52.0
L16	42.7	50.6	38.7	51.0	57.2	50.5	59.8	54.2	54.3
i20	48.6	115.1	88.3	50.8	109.5	72.2	51.1	116.4	85.0
L19	41.8	92.8	57.8	61.4	89.0	49.8	55.6	89.7	63.0
i17	87.3	169.1	88.3	128.7	183.1	154.2	150.0	181.9	150.5
**Mean**	**44.1**	**62.4**	**41.5**	**53.6**	**69.6**	**52.7**	**61.8**	**76.6**	**57.6**

**Table 6:** ILI prediction results for 31 geolocations estimated using MAPE for nowcasting (this week) and forecasting (one and two weeks). Locations with min and max MAPE scores are underlined.

	This Week			One Week			Two Weeks		
	ILI	SM	ILI + SM	ILI	SM	ILI + SM	ILI	SM	ILI + SM
L14	16.81	15.10	18.18	20.90	23.94	11.55	27.46	28.29	13.18
i2	10.88	13.44	11.65	12.39	13.89	12.01	16.24	14.12	12.43
L3	12.96	15.53	17.25	18.15	39.67	12.94	15.89	35.76	26.80
L27	16.75	16.51	9.97	21.59	22.42	12.57	25.47	26.52	14.12
L20	20.13	19.79	17.09	29.51	23.78	27.39	26.68	24.31	17.57
i25	30.92	25.12	19.34	31.88	22.35	27.48	34.77	26.60	34.70
L28	14.36	12.68	11.53	18.71	27.42	14.62	20.84	29.87	13.45
L11	12.30	18.00	10.92	16.48	28.22	16.91	22.27	21.82	22.23
L25	11.43	21.08	6.95	16.95	30.92	8.89	16.39	29.59	12.55
L10	12.34	18.22	17.51	14.37	18.05	18.48	18.80	27.00	16.76
L4	12.34	18.66	10.25	17.38	22.39	17.88	17.83	20.84	19.20
L29	13.87	19.83	12.53	16.13	19.64	14.24	21.39	24.57	21.02
L0	10.37	14.81	9.14	11.74	13.84	10.27	13.58	17.89	13.65
L23	14.67	26.99	11.86	19.32	35.27	17.73	23.86	30.57	22.67
L12	13.96	33.73	15.29	26.28	45.55	30.91	40.91	72.21	42.30
L30	17.50	26.37	16.67	25.96	32.82	19.66	25.14	34.83	29.43
L33	12.30	17.38	10.11	19.34	21.05	12.80	18.93	18.90	15.74
L15	21.32	37.07	18.22	25.91	41.65	33.94	39.65	69.70	38.24
L13	15.57	23.49	14.71	20.10	32.74	23.18	21.90	36.80	26.06
L31	14.17	35.02	12.48	20.84	40.45	21.08	21.39	34.41	25.80
i3	19.67	24.88	22.49	24.34	26.31	25.57	19.61	26.24	28.90
L32	16.25	38.89	20.99	23.80	39.49	27.12	29.24	63.26	36.96
L22	16.36	34.31	13.33	25.30	34.82	27.52	31.78	44.60	37.27
L21	17.50	37.72	16.46	32.36	30.88	29.58	34.16	46.03	38.12
i27	16.16	26.30	17.97	19.03	24.76	19.11	21.96	27.60	22.09
L34	15.74	17.38	9.45	20.99	21.37	13.97	26.00	21.76	17.22
L37	17.63	21.67	16.91	22.82	24.61	18.93	26.28	23.20	21.69
L16	15.08	21.89	13.42	16.89	20.46	16.00	19.14	23.54	18.39
i20	17.08	41.50	28.70	24.68	42.83	29.66	25.63	45.92	39.26
L19	15.99	35.74	19.52	23.39	35.40	22.09	25.66	36.79	28.04
i17	29.39	56.58	31.59	46.21	59.07	47.09	53.29	63.47	55.70
**Mean**	**16.19**	**25.34**	**15.56**	**22.06**	**29.55**	**20.68**	**25.23**	**33.77**	**25.21**

#### Combining ILI and social media predictors

Tables 4–6 show that when LSTM models are trained on both ILI and SM signals the average Pearson correlations are as high as *r* = 0.9 for this week predictions (on average 0.06 higher than ILI only *r* = 0.84), *r* = 0.82 for one week predictions (on average 0.11 higher than ILI only *r* = 0.71), and *r* = 0.74 for two week predictions (on average 0.21 higher than ILI only *r* = 0.53). We observed the highest correlations for locations L14, L11, L4, L12, L10, and L25 and the lowest correlations for international locations—i3, i20, and L20. Note, these international locations have less Twitter data compared to other locations e.g., i3 = 2.73M vs. L14 = 16.43M.

#### Contrasting social media signals with ILI historical data

Tables 4–6 further demonstrate that in case no ILI historical data is available tweet and network features extracted from public social media data can be accurate predictors of location-specific ILI dynamics. This is the case for real-time predictions (nowcasting) as well as one and two week forecasts. More specifically, we show that for some locations, e.g., L3, L27, L28, etc., our LSTM models trained exclusively on social media features can produce predictions up to two weeks in advance with comparable or better accuracy to the models trained on ILI historical data. We observed this for 58% of locations for current week predictions, 75% of locations for one week, and 90% of locations for two week forecasts.

We identified locations with the highest and the lowest performance measured using RMSE, RMSPE, and MAPE metrics. The lowest estimates (the best predictions) were for locations L0, L25, and i2 and the highest estimates—for one international location, i17. Location i17 has the lowest amount of tweets available (0.16M).

**Fig 7:**
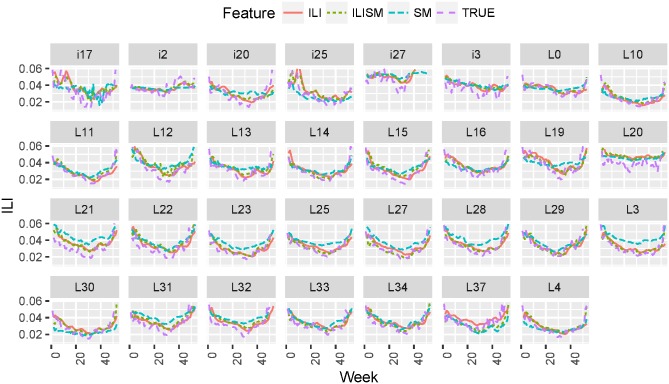
True vs. predicted ILI dynamics one week in advance as a function of time in 2014 for 31 geolocations. We plot true ILI values (True), one week forecasts obtained using social media features only (SM), ILI historical data (ILI), and combined ILI + SM data (ILISM).

**Fig 8:**
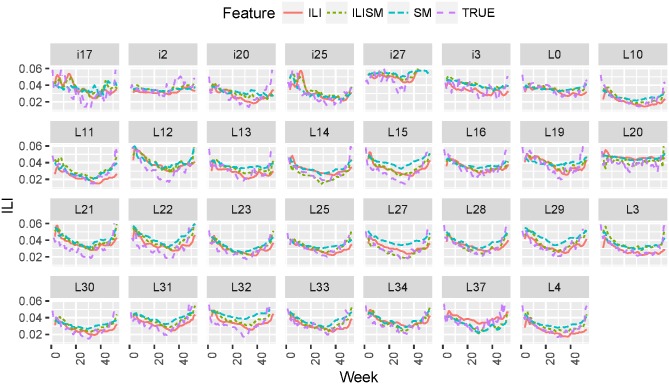
True vs. predicted ILI dynamics two week forecasts as a function of time in 2014 for 31 geolocations. We plot true ILI values (True), two week forecasts obtained using social media features only (SM), ILI historical data (ILI), and combined ILI + SM data (ILISM).

In Figs 7 and 8 we plot true vs. predicted ILI estimates for 2014 season for 31 locations as a function of time. We plot predicted ILI forecasts one and two weeks in advance obtained using LSTM models leaned from ILI historical data only (ILI), social media data only (SM), and combined ILI and social media data (ILISM). We demonstrate that ILI + SM features outperform ILIOnly and SMOnly features; models for one week forecasts are more accurate than models for two week forecasts (lower RMSE, MAPE and higher correlation).

#### Correlating model performance and tweet volume across locations

**Fig 9:**
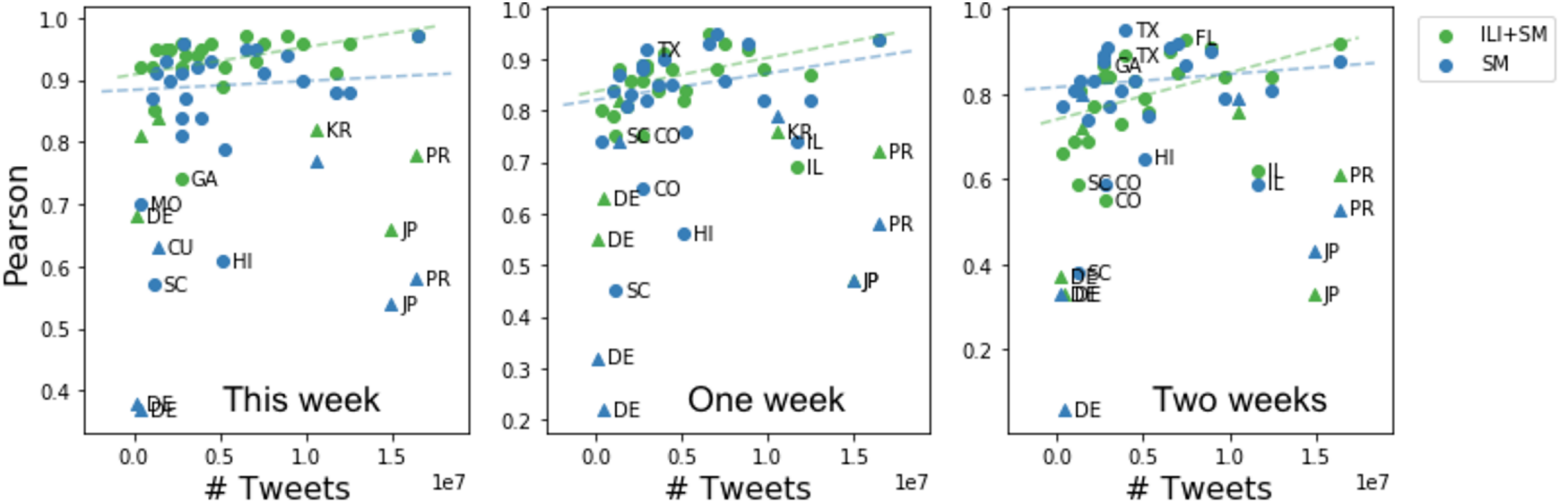
Model performance measured as Pearson correlation between true and predicted ILI dynamics as a function of the number of tweets per location. Predictions are made for the current week, one and two weeks in advance using SMOnly and ILI + SM models. Outlier locations are marked with labels. Trends are shown as dotted lines. International locations are shown as triangles.

**Fig 10:**
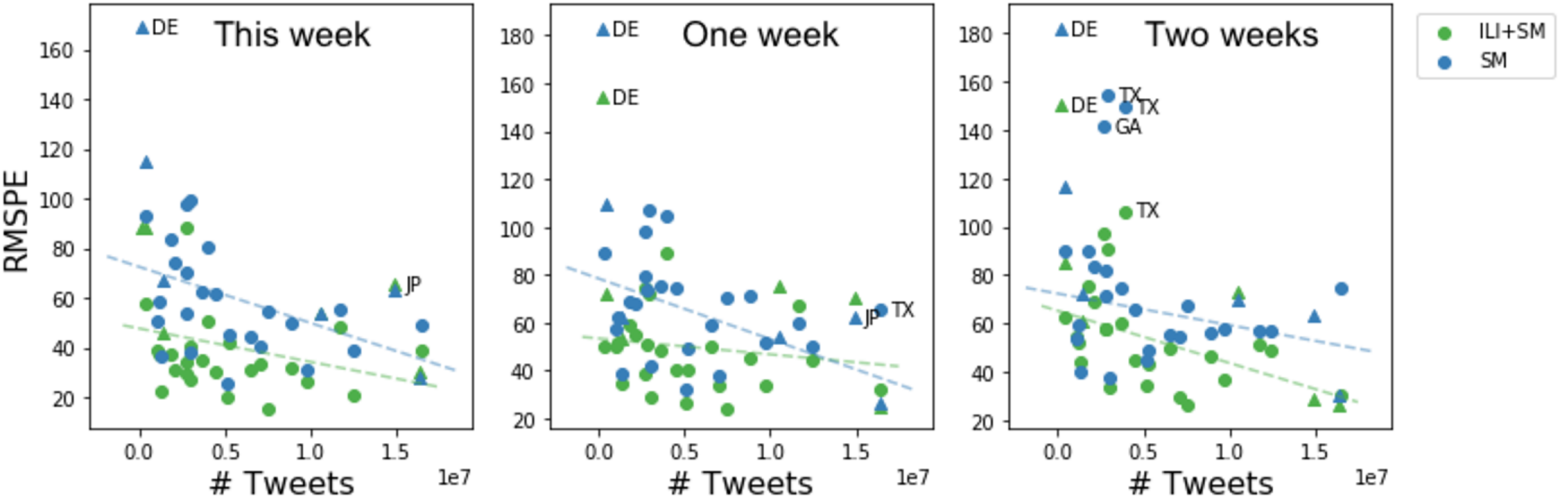
Model performance measured as RMSPE as a function of the number of tweets per location. Predictions are made for the current week, one and two weeks in advance using SMOnly and ILI + SM models. Outlier locations are shown with labels. Trends are shown as dotted lines.

Figs 9 and 10 show how model performance measured using Pearson and RMSPE, respectively, varies across geo-locations depending on the number of tweets available per geo-location. We found that for both neural network models trained on either social media data (SMOnly) or both ILI and social media data (ILI + SM) Pearson between true and predicted ILI dynamics increases with the number of tweets increasing per geolocation. Trends are shown using dotted lines. We observe that most of the outlier locations are international (shown as triangles) e.g., Germany, Puerto Rico, Japan. Similarly, we observe that RMSPE decreases with the number of tweets increasing across geolocations. Outlier locations are mostly international except Texas and Georgia.

## Discussion

Social media disease surveillance has shown significant promise, however, its potential has not been fully evaluated. This work is the first to evaluate the predictive power of previously unexplored signals extracted from social media and use neural network models to forecast location-specific ILI dynamics across multiple geolocations. Unlike earlier work, we contrasted neural network model performance with previously used machine learning approaches, and showed that LSTM models outperform previously used machine learning approaches (SVM, ADABoost). We showed that our models can produce accurate and robust estimates of ILI dynamics up to several weeks in advance.

We also compared the predictive power of location-specific vs. location-independent models, and qualitatively evaluated the value of social media for forecasting ILI activity up to several weeks in advance. We demonstrated that neural network models learned exclusively from social media signals yield comparable or better performance to the models learned from ILI historical data. This suggests that social media signals allow us to track people showing symptoms of influenza (not necessarily confirmed influenza). Thus, social media sources can potentially be used to forecast ILI dynamics for the regions where ILI historical data is not available.

Moreover, neural network models learned from combined ILI and social media signals significantly outperform models that rely solely on ILI historical data, which adds to a great potential trove of alternative public sources for ILI dynamics prediction.

### Modeling influenza awareness vs. infection in social media

Previous work on influenza surveillance that relied on social media sources developed Twitter infection vs. awareness classifiers [6, 7] and filtered flu-related tweets with hand-engineered features [32] to forecast ILI activity [8]. Unlike earlier work, in this study we relied on all tweets produced by the military population in specific geolocations to go beyond influenza-related keywords and tweets, and capture other linguistic predictors e.g., tweets about weather, personal well-being, and travel.

### National vs. local influenza surveillance for targeted populations

The majority of work on ILI surveillance focused on general population in the U.S. and used CDC ILINet as gold standard data [8, 10, 11, 32]. Only limited work developed approaches for local e.g., city-level ILI surveillance [6] and studied ILI dynamics for targeted populations e.g., military populations [33].

Recent work by [34] studied the predictive power of emotions and opinions extracted from user tweets on ILI dynamics. Author relied on social media data to understand the correlation between psychological behavior and health in the military population and the potential for use of social media affects—opinions and emotions extracted from user tweets for prediction of ILI dynamics.

## Conclusions and future work

We presented an approach that uses neural network models and a combination of ILI historical data and social media data to produce more accurate and robust forecasts of location-specific ILI dynamics for targeted populations. We tested our models on a variety of social media signals to predict weekly ILI estimates across 26 U.S. and 5 international geolocations. Our models are capable of predicting weekly ILI dynamics (nowcasting) and forecasting ILI estimates up to several weeks in advance, which can help to overcome a known two week lag-time of CDC reports. Finally, we evaluated the generalizability of predictive models by contrasting model performance across many geo-locations, and analyzed how the predictive power of the model depends on the volume of tweets across locations.

Future work will include exploring image content posted in social media in combination with text and other predictors to forecast ILI dynamics. We are also interested in exploring the predictive power of neural network models and social media signals to model other infectious disease dynamics e.g., ebola, E. coli.
